# Autoimmunity and the Gut

**DOI:** 10.1155/2014/152428

**Published:** 2014-05-13

**Authors:** Andrew W. Campbell

**Affiliations:** The Wellness Center, 23144 Emerson Way, Land O Lakes, FL 34639, USA

## Abstract

Autoimmune diseases have increased dramatically worldwide since World War II. This is coincidental with the increased production and use of chemicals both in industrial countries and agriculture, as well as the ease of travel from region to region and continent to continent, making the transfer of a pathogen or pathogens from one part of the world to another much easier than ever before. In this review, triggers of autoimmunity are examined, principally environmental. The number of possible environmental triggers is vast and includes chemicals, bacteria, viruses, and molds. Examples of these triggers are given and include the mechanism of action and method by which they bring about autoimmunity.

## 1. Introduction

Autoimmune diseases have registered an alarming increase worldwide since the end of the Second World War. This pandemic includes more than 80 autoimmune disorders and increases in both the incidence and prevalence of autoimmune disorders such as Crohn's disease, rheumatoid arthritis, multiple sclerosis, and type I diabetes [1, 2]. In the United States, it is far more commonly found in women and is one of the top 10 leading causes of death in female children and women of all age groups. The National Institutes of Health (NIH) estimates that 23.5 million Americans have an autoimmune disease. In contrast, cancer affects 13 million Americans. Symptoms involve many medical specialties and can affect all body organs (http://www.aarda.org/autoimmune-information-statistics/).

**Figure 1:**
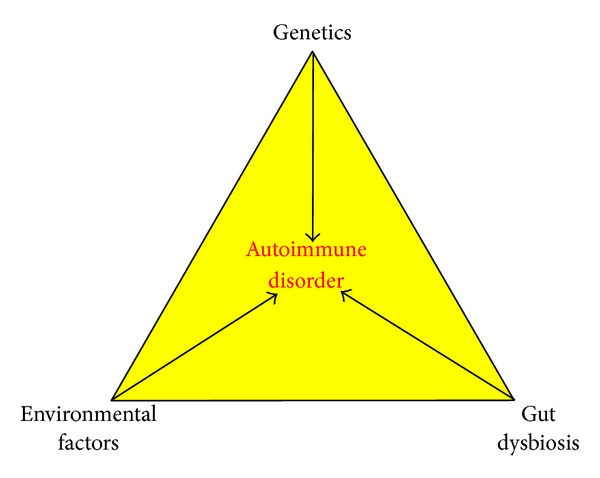
The triangle of autoimmune triggers. Gut dysbiosis and genetic and environmental factors play major roles in the development of autoimmune diseases.

Genetic predisposition, environmental factors (including infections), and gut dysbiosis play major roles in the development of autoimmune diseases (Figure 1). Autoimmunity develops over time, and preclinical autoimmunity precedes clinical disease by many years and can be detected in the peripheral blood in the form of circulating autoantibodies [3]. Initially, symptoms of autoimmune disorders are vague and include fatigue, low-grade fever, muscle and joint aches, and malaise. They usually progress and become debilitating with significant morbidity. Patients are often seen by physicians only after their disease process has become symptomatic, clouding the understanding of the early events leading to disease. The clinician familiar with triggers for autoimmunity can order the right combination of laboratory analyses necessary to elucidate the type and stage of the patient's autoimmune reaction. This in some cases may help the clinician initiate preventive therapies aimed at removing the offending triggers and thereby reverse the progression of the autoimmune disorder with the possibility of eliminating the autoimmune disease.

## 2. Genetics

There are genetic variants that predispose humans to multiple autoimmune diseases and, secondly, multiple genes predispose humans to each disease. The major histocompatibility complex (MHC) is central in mediating inflammatory responses to pathogens. The unique coding or noncoding genetic variations of HLA alleles determine the antigenic responses to self- or non-self-antigens [4]. One of the most common genetic associations with autoimmune disorders is the protein tyrosine phosphatase gene PTPN22 expressed in lymphocytes. The tryptophan allele within PTPN22 has been found in patients with many autoimmune disorders, including type 1 diabetes mellitus, rheumatoid arthritis (RA), systemic lupus erythematosus (SLE), and autoimmune thyroiditis [5, 6]. Cytokines and cytokine receptors are also associated with autoimmune disorders, as can be seen in IL-12/IL-23 pathway in inflammatory bowel disease (IBD), ankylosing spondylitis, and psoriasis [6]. Tumor necrosis factor (TNF) has been linked with autoimmune disorders, notably the TNF-inducible protein A20, which has been associated with RA, psoriasis, and SLE [7]. The importance of CD40 in the maintenance of effector T cell populations in autoimmune diseases has been described in recent studies. Patients with type 1 diabetes (T1D) have increased CD4^lo^CD40^+^ T cells in peripheral blood compared with T2D patients or healthy controls [8]. A polymorphism of CD40 that enhances CD40 signaling is common in patients of Mexican and South American descent; these two groups are known to have increased severity of SLE [9]. In celiac disease (CD), 95% of patients possess the HLA DQ gene; in RA, the HLA variants are DR genes.

There is familial clustering in some autoimmune diseases, suggesting common genetic, developmental, and environmental factors. This has been demonstrated in twin studies with higher disease concordance in monozygotic twins as compared to dizygotic twins. A large population-based survey revealed patients with multiple sclerosis (MS) or rheumatoid arthritis (RA) were more likely to have other autoimmune diseases [10, 11]. However, this concordance rate is only 10–40% for most autoimmune diseases, indicating environmental factors as playing a major role [12].

## 3. Environmental Factors

There are a host of environmental factors that trigger autoimmune disorders, including chemical toxicants, heavy metals, viruses, bacteria, emotional stress, and drugs. For example, adjuvants, such as aluminum hydroxide used in vaccines and medical silicones used in breast implants, can cause an autoimmune disorder known as Shoenfeld's syndrome [13]. A recent study published in the journal Apoptosis demonstrates that hepatitis B vaccine causes liver cell destruction in Hepa1-6 cells. This cell death is attributed to the use of the adjuvant aluminum hydroxide, increasingly identified as a contributing cause of autoimmune disease in immunized patients [14]. Studies show that hepatitis C is almost indistinguishable from autoimmune hepatitis based on biochemical and clinical features. Autoantibodies detected in patients with autoimmune hepatitis are also frequently found in patients with hepatitis C, and both groups of patients suffer from the same immune-mediated symptoms and diseases with chronic hepatitis C [15]. Indeed, 40–70% of patients suffering from hepatitis C also develop at least one extrahepatic inflammatory disorders, including arthritis, vasculitis, and sicca syndrome [16].

Women with silicone breast implants frequently fulfill the diagnostic criteria for autoimmune syndrome induced by adjuvants, known as autoimmune syndrome induced by adjuvants (ASIA). Although the exact mechanism is not known, medical silicones in breast implants are associated with systemic lupus erythematosus, rheumatoid arthritis, vasculitis, and progressive systemic sclerosis [17, 18].

Smoking is a known risk for RA and recent studies have demonstrated that cigarette smoking may induce citrullination of proteins in pulmonary alveolar cells. This is an important finding because antibodies to citrullinated peptides are highly specific for RA as are the HLA associations that are related to the development of these autoantibodies [19, 20].

Infectious agents, including bacteria, viruses, fungi, and parasites, are also known to trigger autoimmune disorders through several mechanisms: molecular mimicry, epitope spreading, standard activation, viral persistence, polyclonal activation, dysregulation of immune homeostasis, and autoinflammatory activation of innate immunity. It is important to note that an infection may not necessarily be the inducer but rather the total burden of infections from childhood on that trigger autoimmunity [21]. Moreover, an infection can amplify an autoimmune disease by either exacerbating an ongoing disorder, including a relapse, or by leading to chronic progressive disease [22].

An example of infectious agents associated with autoimmune disorders is the link between dysregulation of Epstein-Barr virus (EBV) with the occurrence of systemic autoimmune diseases (SADS), a group of connective tissue diseases, including systemic lupus erythematosus (SLE), rheumatoid arthritis (RA), Sjogren's syndrome (SS), and mixed connective tissue disease (MCTD), with overlapping symptoms and antibody development. EBV is an omnipresent infectious virus, affecting approximately 95% of the world's population [23]. It is a DNA virus of the herpes family transmitted in saliva and initially infects epithelial cells in the oro- and nasopharynx. Afterwards, EBV enters the underlying tissues and infects B-cells [24]. In childhood, EBV causes a mild asymptomatic infection; in adolescents, it causes infectious mononucleosis (IM) in 30–70% of cases, and up to 20% of B-cells are infected with EBV [25]. After the first lytic infection, EBV persists in resting memory B-cells for the rest of the patient's life and can switch between an active lytic cycle and a latent state from which it occasionally reactivates, making it a continuous challenge to the patient's immune system [26].

Patients with SLE have an elevated viral load in the peripheral blood mononuclear cells (PBMCs) compared to healthy controls, anywhere from 10 to 40 times higher. The viral load is coupled with disease activity and unrelated to any immunosuppressive medication. A study found an elevated EBV DNA in the serum in 42% of patients compared with 3% of healthy controls [27–29]. Lastly, elevated levels of IgA antibodies to early antigen diffuse (EA/D) were found in 58% of SLE patients versus healthy controls and unrelated to immunosuppressive medication, demonstrating that the antibodies were not due to reactivation of EBV due to a suppressed immune system from medications [30].

In patients with RA, EBV DNA/RNA has been found in PBMCs in saliva, synovial fluid, and synovial membranes, as well as a 10-fold higher frequency of EBC-infected B-cells than in healthy controls [31–33]. This demonstrates widespread lytic EBV infection in RA patients that is also localized in the joints, signifying EBV-infected cells in the synovial inflammation that is characteristic of RA patients [34].

EBV infection has also been demonstrated in SS patients, with EBV-directed antibodies and increased viral load [35, 36]. Patients with SS also have a higher risk for EBV-associated lymphomas [37]. Elevated levels of antibodies to EBNA, VCA, and EA have been found in the serum of SS patients [38, 39]. One study showed IgG antibodies to EA/D in 36% of SS patients compared to 4.5% of healthy controls; these antibodies were not associated with immunosuppressive medication [40].

In conclusion, EBV infection is an example of one of the causal environmental factors in autoimmune disorders. As discussed above, EBV infection can lead to SADS as it can persist in the patient as a latent infection that can occasionally reactivate and cause flares as seen in chronic SADS and other autoimmune disorders.

## 4. Mucosal Immunity

The diet of humans has changed dramatically since the Second World War, especially in industrialized countries and in urban areas. For thousands of generations, humans ate food shortly after harvesting and when it was in season. Meat was occasionally consumed and much of it was caught in the wild. In the past 50 or so years, our foods have undergone a considerable transformation. We have developed new strains of grains, especially in wheat, rice, soy, and corn. In the United States, we use more genetically modified crops than the rest of the world combined. We use chemicals such as pesticides, fungicides, and insecticides for other crops such as fruits and vegetables; we inject dairy cows with hormones passing them on into dairy products; antibiotics, heavy metals, such as arsenic, and hormones are used in concentrated animal feeding operations (CAFO's) which include cattle, hogs, turkey, and chicken; we have chemical ingredients in our foods such as artificial preservatives, colorings, and flavorings; we use artificial sweeteners abundantly, especially in soft drinks; we consume more than twice the amount of salt that we should, leading to cardiovascular disorders and contributing to immune reactions leading to autoimmune disorders [41–44]. Our abundant use of plasticizers such as bisphenol A in food and beverage containers contributes to this overreaching environmental exposure to xenobiotics as well. The widespread use of antibiotics, antacids, proton pump inhibitors, histamine 2 blockers, and other drugs, many of which are available over the counter, adds to what we consume.

Parallel to these dietary changes, there has been a considerable increase in autoimmune diseases such as type 1 diabetes, Crohn's disease, and multiple sclerosis (MS), especially in developed industrialized countries, suggesting a link between diet and autoimmune problems. For example, it has been established that ingestion of gluten leads to gluten enteropathies and vitamin D deficiency has been epidemiologically correlated with a higher risk for autoimmune diseases [45]. Indeed, type 1 diabetes and MS are also linked to low vitamin D levels as are other autoimmune diseases [46].

**Figure 2:**
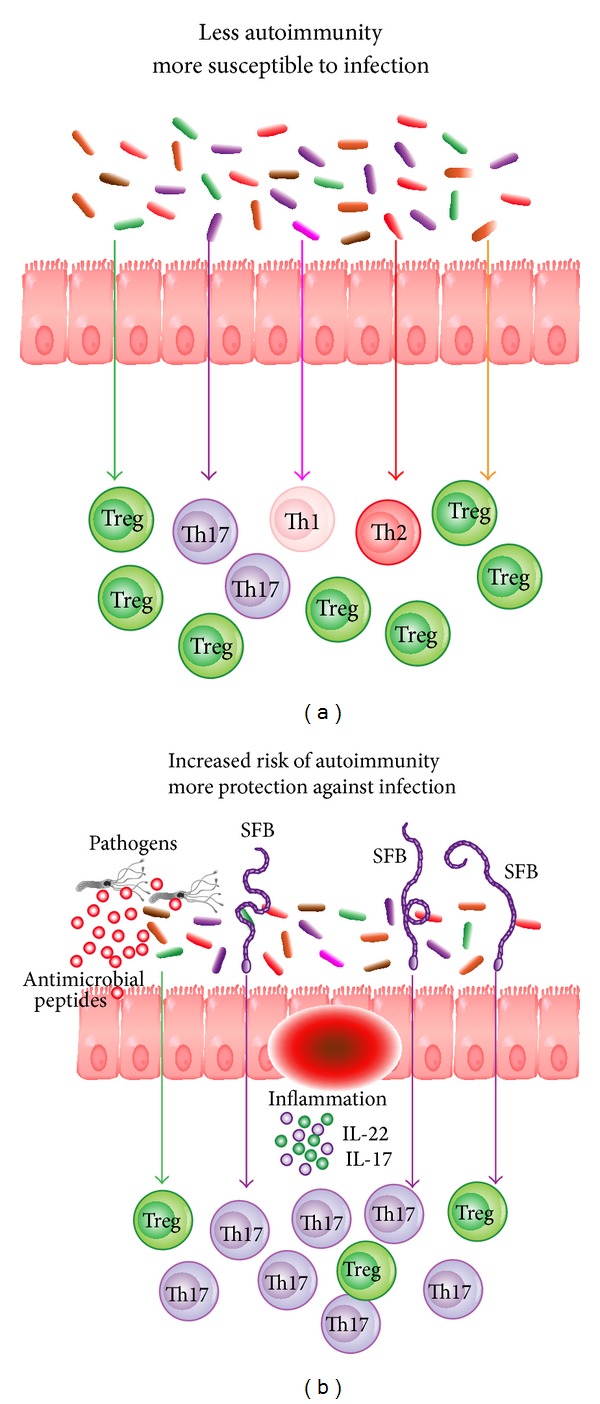
The composition of the intestinal microbiota is involved in the regulation of immune homeostasis. (a) Signals from different components of the microbiota (different colored arrows) regulate different branches of mucosal T cell response (corresponding color immune cells) in the lamina propria. (b) Changes in the composition of commensal bacteria, for example, the introduction of segmented filamentous bacteria (SFB), effect a change in the immune homeostasis, in this case, increasing the signals mediating induction of Th17 cells (purple arrows). This changes the immunological fitness of the individual. In the case of SFB, the increased production of Th17 cell effector cytokines, for example, IL-17 and IL-22, and the consecutive increase in antimicrobial peptide production from epithelial cells (red circles) increase the ability of the host to fight off intestinal infections. However, this increase in proinflammatory cytokines may also render the host more susceptible to chronic autoimmune inflammation. In this way, differences in the composition of the commensal bacteria in the gut may account for differences in individual response in the face of similar environmental challenges. (Adapted from: Ivanov I, Littman D. Segmented filamentous bacteria take the stage. Mucosal Immunology, 3(3):209-12, 2010.).

There are a large number of bacteria in the oral cavity, approximately 10^12^, which include the tongue, teeth, and periodontal tissues. In contrast, the stomach has only 10^3^-10^4^ bacteria and there are 10^8^-10^9^ in the terminal ileum. The greatest number of bacteria is in the large intestine. The majority of these bacteria, approximately 70%, cannot be cultivated by current laboratory microbiological methods [47]. The gut, with a surface area of approximately 200 square meters, is where we come into greatest contact with the outside world and it follows that the gut also has the largest collection of immune cells, consisting of 70% of all lymphoid tissues in the body [48, 49]. It serves to prevent the outgrowth of pathogenic organisms. Recent studies have discussed the human microbiome and its composition in the healthy gut [50, 51]. We carry approximately 1 × 10^(13)^ microorganisms in our gut, more than 10 times the total number of cells in our bodies [52]. The two predominant bacterial phylotypes are* Bacteroidetes *and* Firmicutes *[53]. Interestingly, the number of genes of our intestinal microbiota is 150 times greater than the number of genes in the human genome (Figure 2) [54]. Diet can substantially effect the microbiota. For example, in a diet that is high in fat and protein,* Bacteroides spp*. enterotype predominate, whereas in a diet that is high in carbohydrates,* Prevotella spp. *enterotypes predominate [55].

**Figure 3:**
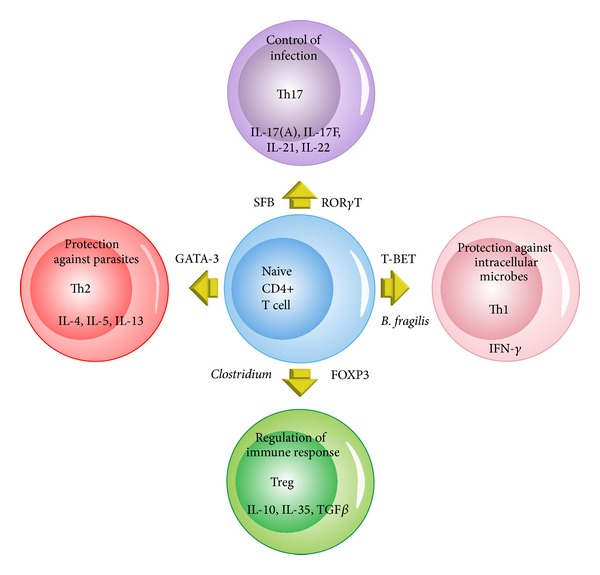
Commensal bacteria induce CD4+ T cell differentiation. Naïve CD4+ T cells can differentiate into four major cell types: Th1, Th2, Tregs, and Th17. The differentiation of each lineage requires the induction of a transcription factor that is unique to each lineage. Once differentiated, each lineage secretes a special set of cytokines. Th1 cells play an important role in eliminating intracellular pathogens while Th2 function to control parasitic infection. The primary role of Th17 is to control infection, while that of Tregs is to regulate immune response. The type of bacteria species that has been shown to induce a particular T cell differentiation pathway is also shown. (Adapted from Wu H, Wu E. The role of gut microbiota in immune homeostasis and autoimmunity. Gut Microbes 3 : 1, 4-14, 2012.).

Mucins are highly glycosylated macromolecules, forming the first barrier between the contents of the gut and epithelial cells. This barrier provides protection for the epithelial cells from direct contact with commensal bacteria and their elements (Figure 3). Changes in either the composition or amount of mucus may lead to inflammatory responses [56]. Secretory IgA is one of the main humoral defense mechanisms ensuring the proper functioning of the mucosal surface barrier. It prevents the adherence of bacteria to mucosal surfaces and the penetration of antigens into the internal environment of the host by specific and nonspecific mechanisms [57, 58]. However, in persons with selective IgA deficiency, the mucosal barrier is deficient and more permeable to immunogens and allergens. Dendritic cells are the main cells that present antigens to the adaptive arm of the mucosal immune system [59]. A mucosal immune response, either one of tolerance or stimulation, depends on the partaking of different populations of dendritic cells responsible for the activation of regulatory T-cells subpopulations [60]. Activation of regulatory T-cells that inhibit the immune response and induce mucosal tolerance is dependent on the production of IL-10 and transforming growth factor-beta [61]. The maturation of dendritic cells is dependent on inducement by pathogenic organisms organisms and this then brings about the activation of effector T cells crucial for clearing infections and the prevention of subsequent infections with the same or related bacteria.

The epithelial cells of the gut have secretory, digestive, and absorptive functions and have receptors to facilitate their participation in immunological processes. The signaling pathways of these cells are highly regulated by pathways and molecules to provide a negative feedback system to avert uncontrolled inflammatory responses [62, 63]. Epithelial cells are the first point of contact for gut bacteria [64]. The epithelial layer of the gut is a major barrier between the host and the environment and is composed of a single layer of interconnected epithelial cells. This layer is reinforced by tight junctions in the paracellular spaces between the epithelial cells. These tight junctions of the epithelial layer of the gut act as a highly regulated entry that open and close depending on signals, such as cytokines and bacterial components from the lumen, lamina propria, and epithelium. Tight junctions are essential to the intestinal diffusion mechanisms [65]. The epithelial cells also make contact with the immune system of the gut and line the lamina propria of the small and large intestines and Peyer's patches which are organized lymphoid tissues. The Peyer's patches are critical for the direct antigen sampling from the gut and are where immune responses are induced and regulated. This is essential for gut health as too little or no bacterial exposure, as in germ-free conditions, can impair immune response, whereas excessive contact with bacteria may cause an increase in proinflammatory immune response. IgA and IgM derived from T-cell dependent and T-cell independent activation of B-cells and their differentiation into immunoglobulin secreting plasma cells are fundamental for the regulation of antigen penetration across the gut [66]. Immune regulation assists the gut to support microbiota and to ensure that effector immune responses are activated as a response to invading pathogens. Studies have shown the importance of Tregs in maintaining tolerance to the microbiota in the gut [67].

## 5. The Gut Microbiota

The gut microbiota can be influenced by several factors: the motility of the gastrointestinal tract (GIT); the intake of pharmaceutical medications, including antacids, antibiotics, and nonsteroidal anti-inflammatory drugs; smoking; the use of alcohol; the GIT transit time; mucosal blood flow; and renal clearance [68, 69]. These factors can lead to the uptake of antigens from the lumen, which play an important role in the pathogenesis of gastrointestinal disorders (Figure 6). The disproportionate uptake of these antigens, coupled with the suppression of immune responsiveness or the failure in immunological tolerance, can lead to immunological reactions both within the gut and in other organs and follow one of two pathways: physiologic transport and pathological transport. Physiologic transport consists of ligand-receptor uptake, antibody uptake, and lastly microfold or M cell transport. Pathological transport is either antigen-specific or -nonspecific. Antigen specific transport via the transcellular of paracellular pathways has the ability to bring about a specific disease.

Examples include celiac disease (CD), gliadin, and allergic gastroenteropathies with casein and beta-lactoglobulin. The antigen nonspecific transport occurs when the tight junction becomes more permeable due to environmental factors which activate inflammatory cascade via transcellular or paracellular pathways [70, 71]. Vojdani in his recent study concluded that “increased antigen uptake in the intestine precedes the onset of many immunologically mediated gastrointestinal diseases” [72]. CD is frequently associated with other autoimmune disorders, in particular type 1 diabetes (T1D) and thyroiditis. This suggests that CD shares some common pathogenic mechanisms with other autoimmune diseases [73]. Genetic studies in patients with CD and T1D have shown gut mucosal barrier dysfunction [74–76]. In CD, we now know that disease-specific autoantibodies are directed against the enzyme transglutaminase 2 (TG2) brought about by gluten-reactive T cells within the celiac lesions, giving rise to glutamic acid (deaminated glutamine) [77–79].

One of the easiest ways to affect human health is through nutrition and diet. This, in turn, is influenced to a significant degree by the gut microbiota. Going from a low fat, plant polysaccharide rich diet to a high fat, high sugar Western diet changed the microbiota in one day in GF mice. There were more members of the Firmicute classes Erysipelotrichi and Bacilli (Enterococcus) and less Bacteroidetes associated with the Western diet. Another notable finding was that there was a significant increase in adiposity in humanized mice fed the Western diet as compared to those fed the low fat plant polysaccharide diet. These are important findings as they demonstrate that the gut microbiome can change over a very short period of time [80].

Recent studies have shown that the colonization of the small intestine in mice with a single commensal microbe, segmented filamentous bacterium (SFB), induced Th17 cells in the lamina propria. SFB are spore-forming Gram-positive bacteria related to the genus* Clostridium *and are found in many species as well as in humans. They are associated with reduced colonization and growth of pathogenic bacteria in the ileum where they are most abundant and adhere tightly to the epithelium. This colonization with SFB resulted in augmented resistance to* Citrobacter rodentium*, an intestinal pathogen, and with increased expression of genes linked with inflammation and antimicrobial defenses [81]. TGF-*β* differentiate Th17 and Treg cells and are defined by the expression of lineage-specific transcription factors ROR*γ*t and Foxp3 [82–86]. Th17 cells are essential mediators of autoimmune diseases, as they have potent inflammatory effects; they have important roles in protection from bacterial and fungal infections, especially at mucosal surfaces, and secrete IL-17, IL-17F, and IL-22. The increased production of Th17 cell effector cytokines, for example, IL-17 and IL-22, and the consecutive increase in antimicrobial peptide production from epithelial cells augment the ability of the host to fight off intestinal infections. At the same time, however, this increase in proinflammatory cytokines may render the host more susceptible to chronic autoimmune inflammation [87].

## 6. The Gut and Rheumatic Disease

Rheumatoid arthritis (RA) is one of the most prevalent systemic autoimmune diseases targeting principally the joints. RA leads to joint deformity, disability, and increased mortality without treatment. It is a multifactorial and complex disease caused by genetic and environmental factors with increased production of self-reactive antibodies and proinflammatory T lymphocytes [88]. In RA there is a prolonged period of autoimmunity with circulating autoantibodies such as rheumatoid factor and anticitrullinated peptide antibodies. This preclinical state may last many years without any clinical signs or symptoms of inflammatory arthritis. However, there is an increase in antibody titers and epitope spreading with elevation in circulating proinflammatory cytokines before the onset of clinical disease. In these situations, environmental factors may be the triggering event for systemic joint inflammation. Microbes from the periodontal tissue, the airways, and the gut microbiota have been implicated [89, 90].

RA has pathogenic disease-specific autoimmunity to citrullinated proteins. Citrullination, a modification of arginine catalized by peptidylarginine deiminase enzymes, has the ability to change the structure, antigenicity, and function of proteins. Porphyromonas gingivalis, a major pathogenic bacterium related to gingivitis, is linked to RA in epidemiological studies and is the only bacterium that expresses endogenous citrullinated proteins [91].

The gut microbiota composition can be changed by antibiotics. Studies have shown that antibiotic use reduced Bacteroides and Bifidobacterium and led to the growth of Campylobacter, Streptococcus, Leuconostoc, or yeasts like* Candida Albicans* in the gut [92].

**Figure 4:**
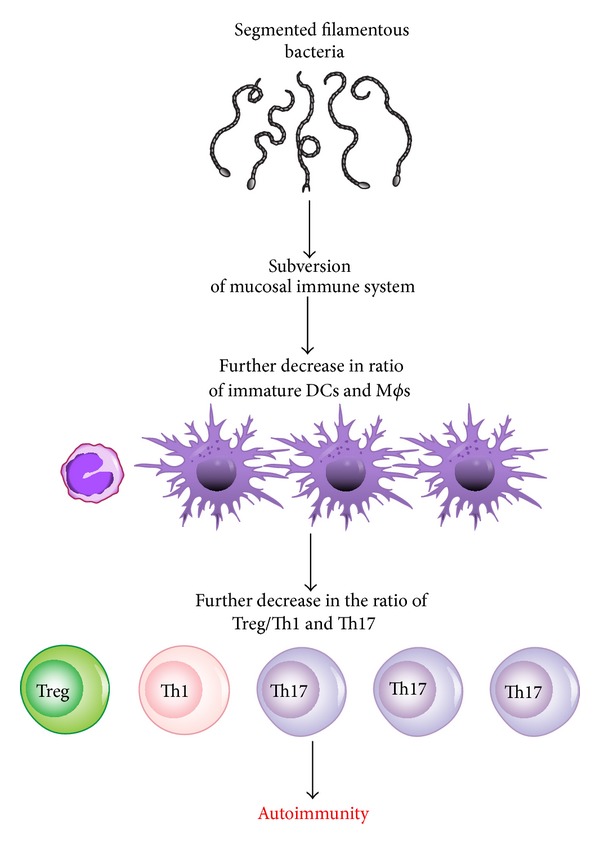
How segmented filamentous bacteria (SFB) can change the ratio between Th17 and Tregs, leading to autoimmunity.

An alteration causing an imbalance in the gut microbiota can change T-cell responses and modulate systemic inflammation. Germ-free mice lack Th17 cells; when the gastrointestinal tract of these mice is colonized with segmented filamentous bacteria (SFB), Th17 cells are induced to accumulate in the lamina propria [81] (Figure 4). Mice raised in germ-free environments are persistently healthy. By introducing specific gut bacterial species, joint inflammation ensues. Treatment with antibiotics in these mice will prevent and negate a rheumatoid arthritis-like phenotype. When the gut of arthritis-prone K/BxN mice gut is colonized with SFB, the inflammatory disease is potentiated by Th17 cells [82]. An imbalance in gut microbiota with predominance of SFB may result in the reduction of functions of Treg cells and a predisposition to autoimmunity. This may affect systemic inflammatory processes and may partially be why there is reduced Treg function in patients with RA. This demonstrates that T cells whose functions are under the control of the gut commensal microbiota can also be the effectors of pathogenesis in autoimmune disorders [83].

A recent study showed that 75% of patients with new onset RA (NORA) carried* Prevotella copri *in their intestinal microbiota. Furthermore, 37.5% of psoriatic arthritis patients also had* Prevotella copri *in their gut compared to 21.4% of healthy controls [93]. This again demonstrates the effects of the environment from the gut microbiota aspect on autoimmune disorders.

Patients with juvenile idiopathic arthritis have been shown to have increased intestinal permeability along with gastrointestinal symptoms, suggesting a role for intestinal changes in the pathogenesis of rheumatic diseases [94]. Arthritis is frequently found in patients with IBD, again suggesting the participation of the gut in immune-mediated rheumatic disorders [95]. IBD is an autoimmune disorder affecting the GI tract in two main forms: Crohn's disease and ulcerative colitis. The phyla of gut microbiota in patients with IBD greatly differ when compared with normal patients [96]. Studies have shown that antibiotics treatment benefits patients as well as animal models of IBD, indicating that bacteria play an important role in the pathogenesis [97]. A recent study has identified the specific microbiota in the dysbiosis of IBD patients. These patients have an overgrowth of proteobacteria and a reduction in Firmicutes and Bacteroides species [98].

Reactive arthritis and autoimmune reactions in joints may be triggered by infections with intestinal microbial pathogens, including Salmonella, Shigella and Yersinia [99]. Antibodies against antigens of certain species of gut bacteria, for example, Proteus, suggest that these bacteria and rheumatoid arthritis have a pathogenic relationship [100]. This parallels the findings in patients with ankylosing spondylitis having increased titers of anti-Klebsiella antibodies suggesting again a bacterial triggering factor [101].

## 7. The Gut and Neuroautoimmunity

**Figure 5:**
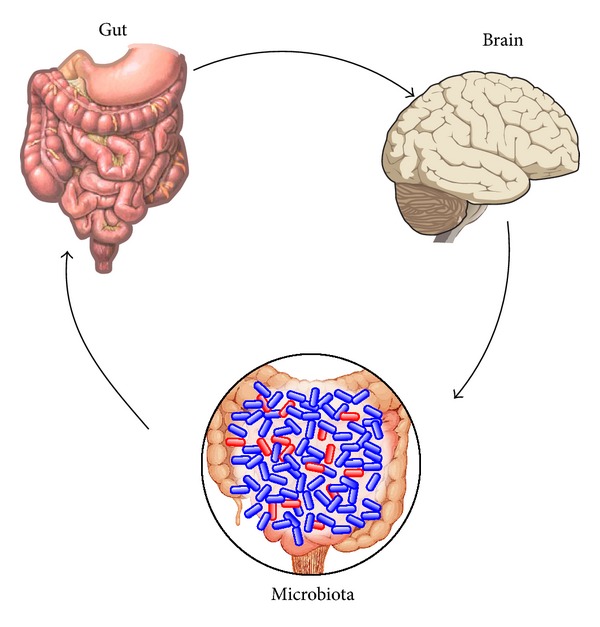
The interconnection of the gut, brain, and microbiota.

**Figure 6:**
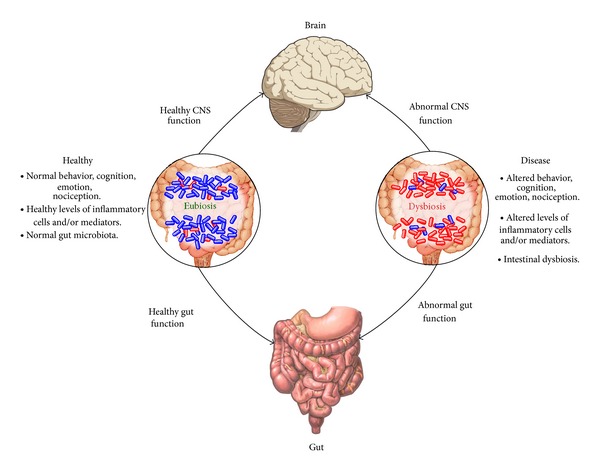
Impact of the gut microbiota on the gut-brain axis in health and disease. It is now generally accepted that a stable gut microbiota is essential for normal gut physiology and contributes to appropriate signaling along the gut-brain axis and, thereby, to the healthy status of the individual (a). On the other hand (b), intestinal dysbiosis can adversely influence gut physiology, leading to inappropriate gut-brain axis signaling and associated consequences for CNS functions and resulting in disease states. Conversely, stress at the level of the CNS can affect gut function and lead to perturbations of the microbiota. (Adapted from: Cryan J, Dinan T. Mind-altering microorganisms: the impact of the gut microbiota on brain and behavior. Nat Rev Neurosci., 13(10):701-12, 2012.).

The gut-brain axis acts as a bidirectional communication between the brain and the gut (Figure 5). The brain modulates gastrointestinal function and the gastrointestinal system is monitored by the brain via neural, immunological, and endocrine mechanisms. The development and function of the enteric nervous system are influenced by the intestinal microbiota [102]. The gastrointestinal system is directly controlled by the enteric nervous systems, the “second brain”. This system consists of more neurons than the spinal cord, mainly in the myenteric and submucosal plexuses. Neuropeptides are able to increase the permeability of tight junctions to macromolecules and thereby modify the function of the mucosal barrier [103, 104].

In adults, chronic stress affects the composition of the gut microbiota with increase of* Bacteroides *spp. and* Clostridium *spp. Coupled with this are increasing levels of IL-6 indicating immune activation [105]. Chronic stress also makes the gut leaky, increasing circulating levels of LPS. Findings of altered intestinal permeability (leaky gut) may play a pathogenic role in patients with depression and their first-degree relatives [106, 107].

Multiple sclerosis (MS) is one of the most frequent and severe demyelinating neurological diseases, mainly affecting young people, eventually leading to their becoming disabled. Increased intestinal permeability in these patients and in their relatives has been reported. MS has also been related to infections with bacteria and viruses [108]. Experimental autoimmune encephalomyelitis (EAE) is the animal model widely used for MS. A study in germ-free mice showed attenuated induction of EAE by myelin oligodendrocyte glycoprotein (MOG) peptide in complete Freund's adjuvant [109]. Another study with mice genetically predisposed to develop EAE showed that when they were housed in germ-free or pathogen-free conditions, they were protected from developing EAE. Once they reached adulthood and had normal gut colonization, the protection was lost [110].

There are increasing numbers of studies demonstrating the importance of the permeability of the gastrointestinal tract to large molecules and how this is linked to the development of various neurodegenerative disorders, including Parkinson's disease (PD). Lewy bodies, the pathological hallmark of Parkinson's disease, were found in intestinal biopsies of patients with PD [108–110].

## 8. The Other Side of the Same Coin

The gut microbiome can also help the host. There are commensal gut bacteria that can ameliorate disease. For example, in immunocompromised mice,* B. fragilis* can lessen the colitis induced by Helicobacter hepaticus via its production of PSA, which stimulates the anti-inflammatory IL-10 production from CD4+ T cells and the downregulating of proinflammatory IL-10 production in the colonic tissues. This, in turn, suppresses disease [111]. In another example, short-chain fatty acids (SCFAs) produced by the gut microbiota interact with G-protein-coupled receptors expressed on immune cells and reduce inflammation in the dextran sulfate sodium (DSS-)induced colitis model [112].

## 9. When Did It All Start?

Bacterial colonization during and shortly after birth plays a major role in the formation of gut microbiota. Factors affecting the communities in this microbiota include premature birth, Caesarean section versus vaginal birth, breast milk versus commercial formula, and many more. For example, premature infants were colonized principally by* C. difficile*. Infants born vaginally were colonized mostly by bacterial communities similar to their mother's vaginal microbiota, including* Lactobacillus*,* Prevotella*, or* Sneathia *spp, whereas Caesarean section born infants were colonized by bacteria found on the skin surface, including* Staphylococcus*,* Corynebacterium, *and* Propionibacterium *species. Formula fed infants had colonization predominantly by* Staphylococci*,* E. coli*,* C. difficile*,* Bacteroides*,* Atopobium, *and* Lactobacilli* [113–117]. Infants delivered via Caesarean section have an increased risk of developing asthma, allergies, and autoimmune disease in later childhood [118, 119]. These are clear demonstrations of the importance of the gut microbiota starting at birth and affecting the patient years later.

## 10. Conclusion

Factors such as genetics, the environment, infections, and the gut microbiota all play a role in the mediation of autoimmune disorders. There have been tremendous recent advances in our understanding of the interplay of these factors. It is clear that the gut microbiota has a profound and long-term effect starting at birth on the host immune system. It is also evident that it plays a significant role in autoimmune diseases both inside and outside the gut. There are still questions that remain to be answered: does the immune system shape the gut microbiota or vice-versa? This complex and dynamic symbiosis needs further elucidation and may help in determining the outcome of autoimmune diseases in patients. The clinician can assist the patient by being aware of the triggers of autoimmune disorders and monitoring immune and autoimmune markers in the peripheral blood, thereby being able to take preventive measures to hopefully avert the progression towards an autoimmune disease.
